# Doing neuroscience in Brazil. The best Brazilian theses in Neuroscience 2007 – a contest sponsored by the IV Brazilian Congress on Brain, Behavior and Emotions

**Published:** 2008

## ABSTRACT-1

**DIFFERENTIATING ATTENTION-DEFICIT/HYPERACTIVITY DISORDER INATTENTIVE AND COMBINED TYPES: A 1H-MAGNETIC RESONANCE SPECTROSCOPY STUDY OF FRONTO-STRIATO-THALAMIC REGIONS**

***Pedro Eugênio Mazzucchi Santana Ferreira, André Palmini, João Rubião Hoefel, Maurício Annès, Paulo Belmonte de Abreu***

Pontifícia Universidade Católica do Rio Grande do Sul. Departamento de Psiquiatria da Faculdade de Medicina da PUCRS e do Programa de Residência do Hospital São Lucas da PUCRS.

### Background:

Few previous investigations have assessed neurobiological substrates for differentiating Attention-Deficit/Hyperactivity Disorder (ADHD) types through the use of modern neuroimaging techniques.

### Methods:

We assessed the neurometabolic profile in frontal-striatal-thalamic right and left regions through 1H-magnetic resonance spectroscopy (1H-MRS) in three groups of subjects (age range: 15-24 years’ old): ADHD inattentive type (ADHD-I; n 10), ADHD combined type (ADHD-C; n =10) and non-ADHD controls (n 12). Compounds that can be visualized with 1H-MRS include N-acetyl-aspartate (NAA), glutamate/glutamine/γ-aminobutyric acid (Glx), creatinine/phosphocreatine(Cr), and choline compounds (Cho).

### Results:

Major differences were detected in metabolic ratios in subjects with ADHD-C compared with those with ADHD-I and controls, even after adjustments for comorbidity. Subjects with ADHD-C showed a lower mI/Cr ratio in the right ventromedial prefrontal cortex (VMPFC) than controls (p 0.004), higher Cho/Cr ratio in the left pulvinar (thalamus posterior) than the ADHD-I group (p 0.02), and higher Glx/Cr ratio in left putamen nucleus than both individuals with ADHD-I and controls (p=0,049). No differences were detected between subjects with ADHD-I and non-ADHD controls. NAA/Cr ratio differed between patients and controls in the left VMPFC only when all ADHD subjects were grouped together (p=0.03).

### Conclusions:

Our findings corroborate previous results suggesting a different neurobiological profile between the two main ADHD subtypes, and suggest that ADHD-C is associated with an energetic deficit in frontal-striatal-thalamic regions. There are two main possibilities to explain our findings in patients with ADHD-C. One is related to interference with neuronal energy-producing mechanisms and the other with neurotransmitter imbalance. Both are probably inter-related within a neurochemical-energetic framework. This neuronal metabolic profile is in line with the ADHD theory of ‘energetic deficit’ in frontal-striatal-thalamic structures. Mioinositol, glutamate/glutamine (Glx) and choline are all related to the energetic neuronal cycle. Furthermore, the reduced mI/Cr in the right VMPFC and increased Cho/Cr in the left pulvinar found in the group with ADHD-C may influence secondary messenger systems bearing upon cyclic-AMP production. The evidence presented here suggests that 1H-MRS of metabolic ratios of mI, Cho, and Glx related to creatinine may differentiate groups of patients with ADHD-C and ADHD-I, without differentiating the inattentive subtype from normal controls. An intriguing explanation for these findings is that these differential metabolic profiles may, at least in part, reveal a greater reduction in the energetic metabolism of frontal-striatal-thalamic structures in the combined subtype of ADHD.

## ABSTRACT-2

**IMPAIRED OCCUPATIONAL AND SOCIAL FUNCTIONING IN SCHIZOPHRENIA LINKED TO DECREASED DORSOLATERAL PREFRONTAL METABOLISM: A PROTON MAGNETIC RESONANCE SPECTROSCOPIC IMAGING STUDY**

**Eloísa Elena Silveira Ferreira, Paulo Belmonte-de-Abreu, André Palmini**

Universidade Católica do Rio Grande do Sul. Unidade Psiquiatra do Hospital São Lucas da PUCRS.

### Introduction:

Reduced frontal N-acetylaspartate (NAA) has been repeatedly found in dorsolateral prefrontal cortex (DLPFC) of chronic schizophrenia and suggests neuronal loss or disfunction. These neuroimaging studies revealed a positive association with reduced NAA and cognitive deficit and severity. Unfortunately, few studies have focused on the association of brain metabolism and impairment in social and occupational functioning.

### Objectives:

To assess the association among DLPFC metabolism by proton magnetic resonance spectroscopic imaging (1H-MRS), with occupational and social handicaps in schizophrenics.

### Methods:

25 subjects with DSM-IV schizophrenia and 12 healthy controls were assessed by 1H-MRS and by selected Scales of Functional Outcomes (SOFAS - Social and Occupational Functioning Assessment Scale), (GAS - Global Assessment Scale), cognitive deficit (WCST - Wisconsin Card Sorting Test) and symptom severity (BPRS- Brief Psychiatric Rating Scale). Differences among means and medians of measures of patients and controls were assessed by Student’s t test and the Mann Whitney test, respectively, confirmed by Covariance Analysis (Ancova), in General Linear Models with SPSS 10.0 software using age, sex, education, age of onset and illness severity as covariates.

### Results:

There was a significant difference in metabolism among schizophrenics and controls regarding several parameters. Schizophrenics displayed lower Right DLPFC metabolism in NAA/Cr ratios (p=0,009). In the Right DLPFC among schizophrenics, NAA/Co ratio (p=0.009) was associated to occupational and social handicap in EAFSO, and NAA/Co ratios (p=0.005) in GAF, and NAA/Cr ratios (p=0.050) were negatively associated to symptom severity (BPRS). On the DLPFC, NAA/Co ratios were negatively associated (p=0.050) to WCST number of completed categories.

### Conclusions:

The study provides fresh evidence about prefrontal metabolism in schizophrenia. The new finding is that lower Right DLPFC metabolism is associated to lower occupational and social functioning in schizophrenia. The additional evidences are (i) lower NAA metabolism (NAA/Cr and NAA/Co ratios) in schizophrenics compared to normal controls, (ii) lower NAA metabolism and functional handicap (measured by EAFSO and GAF, (iii) cognitive deficit (measured by WCST) and (iv) negative association among DLPFC NAA and symptom severity (measured by BPRS). The confirmation of frontal metabolic deficits in schizophrenics compared to normal controls, providing further insight about physiopathology of the illness. This study of metabolic deficits in schizophrenia, if confirmed by additional studies in high risk populations, will strengthen the understanding of neuronal dysfunction and/or neuronal loss, probably preceding schizophrenia onset. Increased brain dysfunction after illness onset would result in decreased coping ability for daily life demands and worse occupational outcomes in schizophrenia.

## ABSTRACT-3

**PREVALENCE OF PSYCHIATRIC COMORBIDITIES IN PATIENTS WITH TEMPORAL LOBE EPILEPSY**

**José Augusto Bragatti, Juliana Bohn Assmann, Vivian Fontana, Clarice Rigotti, Maria Paz Hidalgo, Renata Gomes Londero, Gisele Gus Manfro, Sandra Leistner-Segal, Carolina Machado Torres, Marino Muxfeldt Bianchin**

Universidade Federal do Rio Grande do Sul.

Psychiatric disorders are common in epileptic patients. However, the prevalence of psychiatric comorbidities might be different depending on the group of patients studied and tools used to evaluate neuropsychiatric disorders. Although some studies have evaluated psychiatric comorbidities in epileptic patients, well-controlled studies, using representative patient groups with valid and standardized diagnostic instruments, are still lacking. The objective of this study was to evaluate the prevalence of major psychiatric comorbidities in a series of patients of our population with diagnosis of temporal lobe epilepsy (TLE), using a validated structured clinical interview.

We analyzed 57 patients with TLE, diagnosed according to the 1989 International League Against Epilepsy (ILAE) Classification of Epilepsies and Epileptic Syndromes, using clinical, electroencephalographic, and neuroimaging criteria. All patients were submitted to neuropsychiatric evaluation using the SCID (Structured Clinical interview for DSM-IV axis I Disorders), divided into four major categories: mood disorders, anxiety disorders, psychotic disorders, and drugs and alcohol abuse. Variables studied were age, age of epilepsy onset, duration of epilepsy, gender, family history for epilepsy, seizure frequency, seizure control and psychiatric diagnostics.

There were 21 (36.8%) men and 36 (63.2%) women, with a mean age of 41.8 years, and mean epilepsy duration of 26.1 years. Thirty four patients (59.6%) had major psychiatric comorbidities. Mood disorders, the most commonly occurring, were observed in 22 patients (38.6% of all patients and 64.7% of patients with neuropsychiatric comorbidities). Anxiety disorders were the second most frequent disorder, observed in seven patients (12.3% of all patients and 20.6% of SCID-positive patients). Three patients had psychotic disorders, and another three patients presented drugs or alcohol abuse (5.3% of all patients and 8.8% of patients with neuropsychiatric comorbidities). There were no differences among SCID-positive and SCID-negative patients regarding age, age of epilepsy onset, time of epilepsy duration, gender, family history for epilepsy, seizure frequency, and seizure control.

Our results (psychiatric disorder in 59.6% of patients with TLE) are in line with literature. Most authors have reported psychiatric problems in 19 to 80% of epileptic patients. This large variation is probably attributable to the different patient groups investigated and the even greater variety of diagnostic methods. Due to high prevalence of major psychiatric disorders found in our patients, we believe that centers with psychiatric service dedicated to the evaluation and treatment of psychiatric comorbidities might offer much better clinical care for epileptic patients.

## ABSTRACT-4

**PATTERNS OF SLEEP AND LEVELS OF STRESS IN HOSPITALIZED CHILDREN WHO PLAY OR NOT DURING HOSPITALIZATION**

**Clarisse Potasz, Patrícia G. Ferraz, Luciane Bizari de Carvalho, Lucila F. do Prado, Gilmar F do Prado**

Hospital Infantil Candido Fontoura. Faculdade Metropolitanas Unidas (FMU).

Children are major users of health services but are seldom consulted as health-care consumers. As they are in a state of constant physical and psychosocial development, their ability to understand and cope with different aspects of hospitalization varies with age and developmental stage. Hospitalization can cause stress and children may show immediate or longer-lasting behavioral and emotional problems.

Stress may be considered an adaptive response that enables the organism to cope with threatening stimuli. Stress reactions activate the Hypothalamic-Pituitary-Adrenal (HPA) axis leading to releases of glucocorticoids into the bloodstream. There is a reciprocal interaction between the HPA axis hormone release and sleep, whereby when hormonal levels vary, sleep may also vary. Therefore, changes in sleep may have consequences in the hormonal secretion pattern. Developmentally appropriate play is a useful strategy among other well-known resources that may be used in hospital environments and is suitable for helping children cope with stressful conditions.

The objective of this study was to examine the effect of hospitalization in children regarding sleep parameters (SP) and stress, considering an intervention aimed to lower these levels of stress.

We analyzed SP and urinary cortisol levels (UCL) of 57 children (31 boys) that played (PG) or not (NPG), hospitalized for respiratory diseases in a public pediatric hospital. SP were obtained through sleep logs and UCL were examined in 24-hour urine samples. Children were randomly assigned to play or non-play wards, by doctors responsible for admission who were blind to the conducting of the study.

There were no differences in baseline UCL considering the intervention play, gender, age range, social classification, children’s depression inventory scores and previous experience with hospitalization. Cortisol levels differed significantly (P<0.001) in relation to the intervention play when gender and age range were considered.

In general, younger children slept more during hospitalization than the older individuals in line with their maturity phase. NPG slept significantly more than PG mainly during the day. This was probably due to the use of sleep as a refuge against stress. Boys in our sub groups slept more than girls and showed higher UCL. It is well established that boys and girls show different abilities to cope with stress. PG slept more at night and presented higher rates of sleep efficiency showing that their sleep quality was better than NPG.

In conclusion, our study showed through clinical results that hospitalized children make good use of intervention intended to improve sleep quality and lower levels of stress caused by the disease and the hospitalization process.

## ABSTRACT-5

**PROMNESIC AND ANTIAMNESIC EFFECTS OF A STANDARDIZED EXTRACT FROM MARAPUAMA: ROLES OF ADRENERGIC, DOPAMINERGIC AND SEROTONERGIC RECEPTORS.**

**Adriana Lourenço da Silva, Bárbara da Silva Martins, Juliana G. Ferreira, Domingos S. Nunes, Elaine Elisabetsky**

Universidade Federal do Rio Grande do Sul.

Amazonian peoples use traditional remedies prepared with the roots of Ptychopetalum olacoides (PO), predominantly used by the elderly and those convalescents with CNS disorders and/or those undergoing periods of high physical or mental stress. We had previously shown that a standardized PO ethanol extract (POEE) facilitates long-term memory (LTM) retrieval, and reverses memory deficits in ageing mice using the inhibitory avoidance task. In this study, we showed that memory amelioration was confirmed in aging (14 months) mice presenting memory deficits using the object recognition task. The effect is obtained with the extract given acutely (i.p. 100 mg/kg or p.o. 800 mg/k). POEE (50 and 100 mg/kg/kg, ip) was also effective in reversing the acetylcholine antagonist scopolamine induced impairment in all three memory phases, and the memory consolidation deficit induced by the glutamate antagonist MK-801. Regarding memory phases, in this study we showed that POEE facilitates short-term memory (STM; 3 h after training) acquisition, consolidations and retrieval as well as long-term memory (LTM, 24 h after training) retrieval when administered intraperitoneally (50 e 100 mg/kg) or orally (800 and 1000 mg/kg). By using specific antagonists we also verified that beta-adrenergic and D1 dopamine receptors are required for the facilitatory effects of POEE on STM in all three memory phases, as well as for LTM retrieval. Moreover, POEE promnesic effects on short-term (in all three memory phases) and long-term (retrieval) memories are increased by 5HT2A, but not 5HT1A, serotonin antagonists (spiperone and pindolol, respectively). These results are in agreement with previous data suggesting the interaction of POEE with various neurotransmitter systems, and also in accordance with the therapeutic properties alleged by traditional users of Marapuama based home-made remedies. Considering the results of this study, the anticholinesterase and antioxidant properties previously identified for POEE and its traditional use, this study corroborates the significant potential of this species as a source for drug development in the context of conditions associated with cognitive deficit, including ageing, and Alzheimer disease.

## ABSTRACT-6

**NEUROLEPTIC DRUGS REVERT THE CONTEXTUAL FEAR CONDITIONING DEFICIT PRESENTED BY SPONTANEOUSLY HYPERTENSIVE RATS: A POTENTIAL ANIMAL MODEL OF EMOTIONAL CONTEXT PROCESSING IN SCHIZOPHRENIA?**

**Mariana Bendlin Calzavara, Wladimir Agostini Medrano, Raquel Levin, Sonia Regina Kameda, Monica Levy Andersen, Sergio Tufik, Regina Helena Silva, Roberto Frussa-Filho, Vanessa Costhek Abílio**

Universidade Federal de São Paulo.

The ability to identify emotionally salient information in the environment and form an appropriate and rapid behavioral response is critical to survival. Different psychiatric populations, including schizophrenic, bipolar disorder and attention deficit/hyperactivity disorder (ADHD) patients, present abnormalities in emotion processing.

In this respect, impaired recognition of facial emotion has been demonstrated in schizophrenia, bipolar disorder and ADHD patients. A previous study showed that spontaneously hypertensive rats (SHR), a putative animal model of ADHD, present reduced contextual fear conditioning (CFC), the most common paradigm used to study the biological basis of emotion. The aim of the present study was to characterize the deficit in CFC presented by SHR. Adult male normotensive Wistar rats (NWR) and SHR were submitted to the CFC task.

Sensitivity of the animals to shock, and CFC performance after repeated exposure to the task or repeated training sessions were investigated. We also evaluated the effects of increased foot shock intensity on the CFC. Pharmacological characterization consisted of the evaluation of the effects of the following drugs administered previous to the acquisition of the CFC: pentylenetetrazole (anxiogenic) and chlordiazepoxide (anxiolytic); methylphenidate and amphetamine (used for ADHD); lithium, lamotrigine, carbamazepine and valproic acid (mood stabilizers); haloperidol, ziprasidone, risperidone, quetiapina, amisulpride, clozapine (neuroleptic drugs); metoclopramide and SCH23390 (dopamine antagonists without antipsychotic properties); and ketamine (a psychotomimmetic). The effects of paradoxical sleep deprivation (which worsens psychotic symptoms) and performance on a latent inhibition protocol (an animal model of schizophrenia) were also verified. No differences in the sensitivity to the shock were observed. The repeated exposure to the CFC task, repeated training of this task and increased shock intensity did not modify the deficit in CFC presented by SHR.

Considering pharmacological treatments, only the neuroleptic drugs reversed this deficit. This deficit was potentiated by all pro-schizophrenia manipulations: amphetamine and ketamine administration, and paradoxical sleep deprivation. Finally, a deficit in latent inhibition was also presented by SHR. These findings suggest that the deficit in CFC presented by SHR could represent a useful animal model to study abnormalities in emotional context processing related to schizophrenia.

